# Intraoperative model to flowmetry measurement from coronary-coronary saphenous vein graft bypass

**DOI:** 10.1186/1749-8090-8-S1-P115

**Published:** 2013-09-11

**Authors:** LA Dallan, LAF Lisboa, OA Mejia, F Platania, F Gaiotto, A Milanez, JC Iglésias, FB Jatene

**Affiliations:** 1 Cardiovascular Surgery Department, Heart Institute (InCor), Hospital das Clínicas da Faculdade de Medicina da Universidade de São Paulo, São Paulo, Brazil

## Background

Late hemodynamics studies demonstrated the possibility of saphenous vein graft patency between coronary arteries that received sequential bridges, even when these grafts were completely occluded at its origin. The aim of this study was to evaluate the flowmetry of coronary-coronary saphenous vein graft.

## Methods

We measured intraoperative grafts flowmetry in three patients with important retrograde flow from the coronary arteries throught saphenous vein grafts, verified after distal anastomosis. The surgeries were performed off pump. After the revascularization was completed, in the intraoperative model, we partially clamped the aorta, including both proximal veins anastomosis, and measured the flowmetry from one graft to the other. We verified the flow from vein to vein, with good myocardial perfusion.

## Results

Flows and pulsatile index (PI) of the patients were: Patient 1- Flow of the circumflex coronary artery to the right coronary artery-22ml/ min,PI-4.2.Patient 2 - Flow of the right coronary artery to the left circumflex artery-54ml/min, PI-10.9. Patient 3 - Flow of the circumflex artery to diagonal artery- 27 ml /min, PI-3.1.

## Conclusion

This is an evidence-based test that shows the possibility of obtaining flow between the coronary arteries through the bypass grafts. In rare cases when patient´s grafts available are not long enough to reach the usual proximal sites of arterial blood flow (ascending aorta or to make a composed graft), these evidences offer to the surgeon an alternative arterial blood source.

